# *catena*-Poly[[bis­(μ-5-bromo­pyridine-3-carboxyl­ato-κ^2^ *O*:*O*′)dicopper(II)]-bis­(μ-5-bromo­pyridine-3-carboxyl­ato)-κ^3^ *O*,*O*′:*N*;κ^3^ *N*:*O*,*O*′]

**DOI:** 10.1107/S160053681004242X

**Published:** 2010-10-23

**Authors:** Paul DeBurgomaster, Jon Zubieta

**Affiliations:** a Department of Chemistry, Syracuse University, Syracuse, New York 13244, USA

## Abstract

The title compound [Cu_2_(C_6_H_3_BrNO_2_)_4_]_*n*_, forms sheets in the *bc* plane. The structure features the dinuclear paddle-wheel cage motif common to copper(II) carboxyl­ates. The polymeric structure is achieved through bridging between binuclear units by the pyridyl donors of two of the four carboxyl­ates of the cage. Each cage engages in axial bonding at each copper atom to a pyridyl nitro­gen donor and extends two 5-bromo­pyridine-3-carboxyl­ate groups to bridge to adjacent binuclear sites in the *bc* plane. Each cage is linked to four adjacent cages in the plane. The intra­dimer Cu⋯Cu distance is 2.6465 (5) Å. The remaining 5-bromo­pyridine-3-carboxyl­ate groups project into the inter­lamellar domain and inter­digitate in pairs from each neighboring layer.

## Related literature

For a general review of copper(II) carboxyl­ates, see: Doedens (1976 ▶). For polynuclear copper carboxyl­ates with the [Cu_2_(O_2_C*R*)_4_] core, see: Agterberg *et al.* (1997 ▶); Valentine *et al.* (1974 ▶); Yamanaka *et al.* (1991 ▶). For the preparation of copper coordination polymers under hydro­thermal conditions, see: Lu (2003 ▶). For general discussion of hydro­thermal methods, see: Gopalakrishnan (1995 ▶); Zubieta (2004 ▶). 
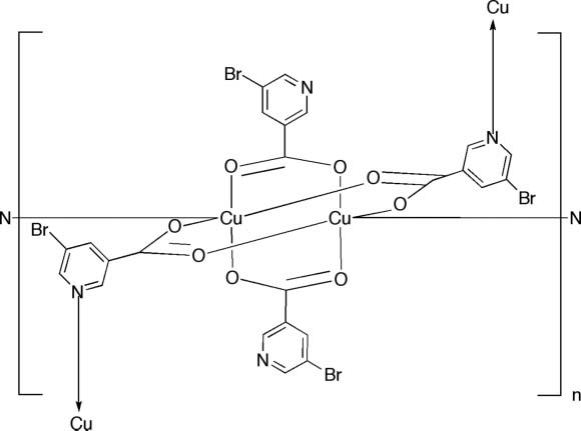


## Experimental

### 

#### Crystal data

[Cu_2_(C_6_H_3_BrNO_2_)_4_]
*M* *_r_* = 931.11
Monoclinic, 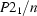

*a* = 11.1390 (12) Å
*b* = 11.5866 (13) Å
*c* = 12.6325 (14) Å
β = 115.432 (2)°
*V* = 1472.4 (3) Å^3^
*Z* = 2
Mo *K*α radiation
μ = 6.93 mm^−1^
*T* = 90 K
0.35 × 0.30 × 0.27 mm

#### Data collection

Bruker APEX CCD area-detector diffractometer
Absorption correction: multi-scan (*SADABS*; Bruker, 1998 ▶) *T*_min_ = 0.196, *T*_max_ = 0.256
14281 measured reflections
3571 independent reflections
3218 reflections with *I* > 2σ(*I*)
*R*_int_ = 0.022

#### Refinement

*R*[*F* ^2^ > 2σ(*F* ^2^)] = 0.021
*wR*(*F* ^2^) = 0.054
*S* = 1.05
3571 reflections
190 parameters
H-atom parameters constrained
Δρ_max_ = 0.77 e Å^−3^
Δρ_min_ = −0.51 e Å^−3^

### 

Data collection: *SMART* (Bruker, 1998 ▶); cell refinement: *SAINT* (Bruker, 1998 ▶); data reduction: *SAINT*; program(s) used to solve structure: *SHELXS97* (Sheldrick, 2008 ▶); program(s) used to refine structure: *SHELXL97* (Sheldrick, 2008 ▶); molecular graphics: *DIAMOND* (Brandenburg & Putz, 1999 ▶); software used to prepare material for publication: *SHELXTL* (Sheldrick, 2008 ▶).

## Supplementary Material

Crystal structure: contains datablocks I, global. DOI: 10.1107/S160053681004242X/rn2072sup1.cif

Structure factors: contains datablocks I. DOI: 10.1107/S160053681004242X/rn2072Isup2.hkl

Additional supplementary materials:  crystallographic information; 3D view; checkCIF report

## Figures and Tables

**Fig. 1.**
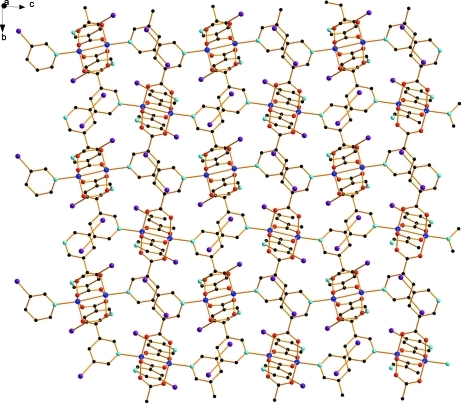
A view of the two-dimensional structure of [Cu2(O2CC5H3NBr)4] in the bc plane. Color scheme as for Fig. 2.

**Fig. 2.**
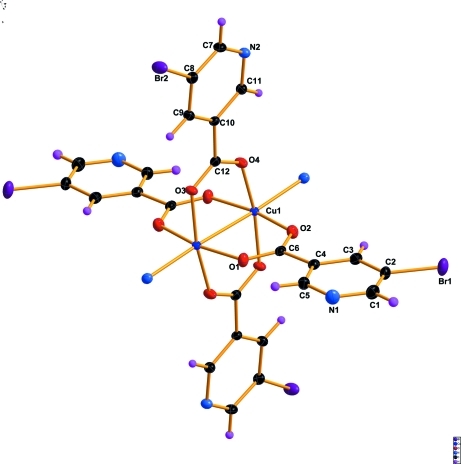
An ORTEP view of the dinuclear paddle-wheel cage building block of the title compound, showing the atom-labeling scheme for the asymmetric unit and 50% displacement ellipsoids. Color scheme: Cu, blue; Br, maroon; O, red; N, light blue; C, black; H, pink.

**Fig. 3.**
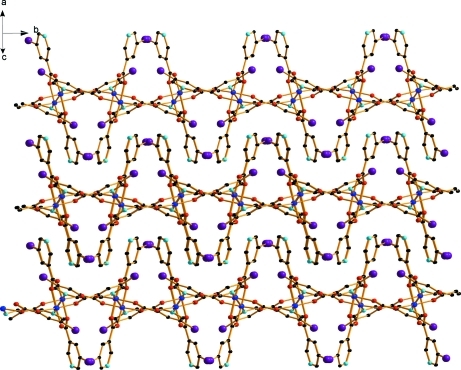
A view of the packing showing the interdigitation of the pendant 5-bromo-2-pyridyl groups. Color scheme as for Fig. 2.

## supplementary crystallographic information

### Comment

The dinuclear *paddle-wheel* cage structure of copper(II) carboxylates is well established [Doedens (1976)]. Polymeric structures incorporating this core can be obtained using ligands capable of bridging between the dinuclear units [Agterberg, *et al.* (1997); Valentine, *et al.* (1974); Yamanaka, *et al.* (1991)]. Since hydrothermal methods are most effective for the preparation and crystallization of organic-inorganic coordination polymers [Gopalakrishnan (1995); Zubieta (2004)], the crystal engineering of copper-containing materials under these conditions has witnessed considerable contemporary attention [Lu (2003)]. In the course of our investigations of Cu(II)-ligand substructures in complex metal oxide hybrid materials, the two-dimensional material [Cu_2_(O_2_CC_5_H_3_NBr)_4_] was isolated.

As shown in Fig. 1, the title compound is two-dimensional, forming sheets oriented parallel to the crystallographic *bc* plane. The fundamental building block of these sheets is the dinuclear *paddle-wheel* cage structure [Cu_2_(O_2_CR)_4_], shown in Fig. 2. The four oxygen donors of the basal plane about the crystallographically unique copper site exhibit Cu—O distances in the range of 1.959 (1)Å to 1.985 (1) Å. There is a crystallographic inversion center at the mid-point of the CuLCu vector relating the two halves of the cage. The Cu···Cu distance is 2.6465 (5) Å.

The cages are linked into the two-dimensional network through the pyridylnitrogen donors of two of the 5-bromopyridine-3-carboxylato ligands, with a copper-axial nitrogen distance of 2.160 (2) Å. The connectivity pattern links each [Cu_2_(O_2_CR)_4_] cage to four neighboring cages to provide the 2-D extension. Two 5-bromopyridine-3-carboxylato ligands of each cage are pendant and project from either face of the polymeric sheets into the interlamellar domains (Fig. 3). These projecting groups interdigitate in pairs with those of neighboring sheets to provide a relatively densely packed arrangement of sheets.

### Experimental

A solution containing Cu(II) acetate hydrate (0.201 g, 1.01 mmol), 5-bromo-2-pyridylcarboxylic acid (0.102, 0.50 mmol), methanol (5.00 ml, 123.56 mmol), and DMF (5.00 ml, 64.58 mmol), in the mole ratio 2.02:1.00:247:129 was stirred briefly before transfer to a 20 ml glass vial. The capped vial was heated at 75 *^o^*C for 72 h. Green blocks of the title compound, suitable for X-ray diffraction, were isolated in 50% yield. Initial and final pH values of 4.0 and 4.0, respectively, were recorded. Anal. Calcd. for C_12_H_6_Br_2_CuN_2_O_4_:*C*, 30.9; H, 1.29; N, 6.01. Found: C, 29.7; H, 1.55; N, 5.93.

### Refinement

All hydrogen atoms were discernable in the difference Fourier map. The hydrogen atoms were placed in calculated positions with C—H = 0.95 Å and included in the riding model approximation with *U*_iso_(H) = 1.2*U*_eq_(C).

### Crystal data

**Table:**

[Cu_2_(C_6_H_3_BrNO_2_)_4_]	*F*(000) = 892
*M**_r_* = 931.11	*D*_x_ = 2.100 Mg m^−^^3^*D*_m_ = 2.09 (2) Mg m^−^^3^*D*_m_ measured by flotation
Monoclinic, *P*2_1_/*n*	Mo *K*α radiation, λ = 0.71073 Å
Hall symbol: -P 2yn	Cell parameters from 3266 reflections
*a* = 11.1390 (12) Å	θ = 2.4–28.3°
*b* = 11.5866 (13) Å	µ = 6.93 mm^−^^1^
*c* = 12.6325 (14) Å	*T* = 90 K
β = 115.432 (2)°	Block, blue
*V* = 1472.4 (3) Å^3^	0.35 × 0.30 × 0.27 mm
*Z* = 2	

### Data collection

**Table:**

Bruker APEX CCD area-detector diffractometer	3571 independent reflections
Radiation source: fine-focus sealed tube	3218 reflections with *I* > 2σ(*I*)
graphite	*R*_int_ = 0.022
Detector resolution: 512 pixels mm^-1^	θ_max_ = 28.1°, θ_min_ = 2.0°
φ and ω scans	*h* = −14→14
Absorption correction: multi-scan (*SADABS*; Bruker, 1998)	*k* = −15→14
*T*_min_ = 0.196, *T*_max_ = 0.256	*l* = −15→16
14281 measured reflections	

### Refinement

**Table:**

Refinement on *F*^2^	Primary atom site location: structure-invariant direct methods
Least-squares matrix: full	Secondary atom site location: difference Fourier map
*R*[*F*^2^ > 2σ(*F*^2^)] = 0.021	Hydrogen site location: inferred from neighbouring sites
*wR*(*F*^2^) = 0.054	H-atom parameters constrained
*S* = 1.05	*w* = 1/[σ^2^(*F*_o_^2^) + (0.0258*P*)^2^ + 1.3076*P*] where *P* = (*F*_o_^2^ + 2*F*_c_^2^)/3
3571 reflections	(Δ/σ)_max_ = 0.001
190 parameters	Δρ_max_ = 0.77 e Å^−^^3^
0 restraints	Δρ_min_ = −0.51 e Å^−^^3^

### Special details

**Table:**

Geometry. All e.s.d.'s (except the e.s.d. in the dihedral angle between two l.s. planes) are estimated using the full covariance matrix. The cell e.s.d.'s are taken into account individually in the estimation of e.s.d.'s in distances, angles and torsion angles; correlations between e.s.d.'s in cell parameters are only used when they are defined by crystal symmetry. An approximate (isotropic) treatment of cell e.s.d.'s is used for estimating e.s.d.'s involving l.s. planes.
Refinement. Refinement of *F*^2^ against ALL reflections. The weighted *R*-factor *wR* and goodness of fit *S* are based on *F*^2^, conventional *R*-factors *R* are based on *F*, with *F* set to zero for negative *F*^2^. The threshold expression of *F*^2^ > σ(*F*^2^) is used only for calculating *R*-factors(gt) *etc*. and is not relevant to the choice of reflections for refinement. *R*-factors based on *F*^2^ are statistically about twice as large as those based on *F*, and *R*- factors based on ALL data will be even larger.

### Fractional atomic coordinates and isotropic or equivalent isotropic displacement parameters (Å^2^)

**Table:**

	*x*	*y*	*z*	*U*_iso_*/*U*_eq_	
Br1	−0.18056 (2)	0.76143 (2)	−0.11231 (2)	0.02559 (7)	
Br2	0.84249 (2)	0.868086 (18)	0.603257 (19)	0.02064 (6)	
Cu1	0.56886 (2)	1.02600 (2)	0.11208 (2)	0.01009 (6)	
O1	0.40781 (14)	0.98221 (13)	0.12844 (14)	0.0179 (3)	
O2	0.29176 (14)	0.93567 (13)	−0.06153 (13)	0.0174 (3)	
O3	1.00043 (14)	1.31393 (12)	0.57312 (13)	0.0153 (3)	
O4	0.88355 (15)	1.36151 (12)	0.38361 (13)	0.0176 (3)	
N1	0.08987 (19)	0.89646 (18)	0.19994 (17)	0.0232 (4)	
N2	0.70137 (17)	1.05035 (15)	0.29489 (15)	0.0142 (3)	
C1	−0.0161 (2)	0.8474 (2)	0.1151 (2)	0.0214 (5)	
H1	−0.0875	0.8233	0.1320	0.026*	
C2	−0.0258 (2)	0.83017 (19)	0.00277 (19)	0.0176 (4)	
C3	0.0770 (2)	0.86282 (17)	−0.0242 (2)	0.0161 (4)	
H3	0.0718	0.8518	−0.1006	0.019*	
C4	0.1886 (2)	0.91256 (18)	0.06474 (19)	0.0146 (4)	
C5	0.1900 (2)	0.92849 (19)	0.17425 (19)	0.0181 (4)	
H5	0.2657	0.9639	0.2340	0.022*	
C6	0.30570 (19)	0.94647 (17)	0.04201 (19)	0.0139 (4)	
C7	0.7262 (2)	0.96880 (18)	0.37741 (18)	0.0146 (4)	
H7	0.6806	0.8971	0.3561	0.018*	
C8	0.8166 (2)	0.98675 (17)	0.49263 (18)	0.0137 (4)	
C9	0.8847 (2)	1.09039 (17)	0.52675 (18)	0.0138 (4)	
H9	0.9470	1.1034	0.6056	0.017*	
C10	0.85843 (19)	1.17443 (17)	0.44120 (18)	0.0116 (4)	
C11	0.76676 (19)	1.15074 (17)	0.32653 (18)	0.0132 (4)	
H11	0.7500	1.2083	0.2683	0.016*	
C12	0.92049 (19)	1.29269 (17)	0.46889 (18)	0.0124 (4)	

### Atomic displacement parameters (Å^2^)

**Table:**

	*U*^11^	*U*^22^	*U*^33^	*U*^12^	*U*^13^	*U*^23^
Br1	0.01981 (11)	0.03107 (13)	0.02547 (13)	−0.01189 (9)	0.00931 (10)	−0.00522 (10)
Br2	0.03049 (12)	0.01399 (11)	0.01496 (11)	−0.00414 (8)	0.00738 (9)	0.00332 (8)
Cu1	0.01025 (11)	0.00862 (12)	0.00980 (12)	0.00020 (8)	0.00280 (9)	0.00007 (9)
O1	0.0152 (7)	0.0211 (8)	0.0180 (8)	−0.0049 (6)	0.0078 (6)	−0.0028 (6)
O2	0.0140 (7)	0.0229 (8)	0.0163 (8)	−0.0013 (6)	0.0073 (6)	0.0000 (6)
O3	0.0183 (7)	0.0113 (7)	0.0127 (7)	−0.0035 (6)	0.0033 (6)	−0.0016 (6)
O4	0.0220 (8)	0.0105 (7)	0.0139 (8)	−0.0030 (6)	0.0015 (6)	0.0012 (6)
N1	0.0233 (10)	0.0302 (11)	0.0186 (10)	−0.0033 (8)	0.0113 (8)	0.0015 (8)
N2	0.0163 (8)	0.0120 (8)	0.0130 (9)	−0.0012 (6)	0.0049 (7)	−0.0006 (7)
C1	0.0197 (10)	0.0245 (11)	0.0239 (12)	−0.0031 (9)	0.0130 (9)	0.0016 (9)
C2	0.0142 (9)	0.0171 (10)	0.0201 (11)	−0.0033 (8)	0.0060 (8)	−0.0010 (9)
C3	0.0176 (10)	0.0140 (10)	0.0184 (11)	0.0002 (8)	0.0092 (8)	0.0003 (8)
C4	0.0143 (9)	0.0124 (9)	0.0175 (10)	0.0008 (7)	0.0073 (8)	0.0024 (8)
C5	0.0169 (10)	0.0190 (11)	0.0170 (11)	−0.0025 (8)	0.0060 (8)	0.0001 (8)
C6	0.0132 (9)	0.0090 (9)	0.0197 (11)	0.0008 (7)	0.0071 (8)	0.0017 (8)
C7	0.0168 (10)	0.0119 (9)	0.0151 (10)	−0.0038 (7)	0.0068 (8)	−0.0024 (8)
C8	0.0174 (9)	0.0116 (9)	0.0125 (10)	0.0005 (7)	0.0068 (8)	0.0038 (8)
C9	0.0145 (9)	0.0143 (10)	0.0115 (10)	−0.0005 (7)	0.0045 (8)	−0.0016 (8)
C10	0.0130 (9)	0.0094 (9)	0.0127 (10)	0.0000 (7)	0.0056 (7)	−0.0011 (7)
C11	0.0142 (9)	0.0114 (9)	0.0132 (10)	−0.0007 (7)	0.0053 (8)	0.0013 (7)
C12	0.0125 (9)	0.0107 (9)	0.0154 (10)	0.0004 (7)	0.0072 (8)	−0.0007 (8)

### Geometric parameters (Å, °)

**Table:**

Br1—C2	1.888 (2)	C1—C2	1.390 (3)
Br2—C8	1.892 (2)	C1—H1	0.9500
Cu1—O1	1.9588 (14)	C2—C3	1.380 (3)
Cu1—O2^i^	1.9665 (14)	C3—C4	1.393 (3)
Cu1—O4^ii^	1.9726 (14)	C3—H3	0.9500
Cu1—O3^iii^	1.9852 (14)	C4—C5	1.389 (3)
Cu1—N2	2.1595 (18)	C4—C6	1.504 (3)
Cu1—Cu1^i^	2.6465 (5)	C5—H5	0.9500
O1—C6	1.261 (3)	C7—C8	1.384 (3)
O2—C6	1.255 (3)	C7—H7	0.9500
O3—C12	1.257 (2)	C8—C9	1.387 (3)
O4—C12	1.259 (2)	C9—C10	1.389 (3)
N1—C1	1.334 (3)	C9—H9	0.9500
N1—C5	1.340 (3)	C10—C11	1.395 (3)
N2—C11	1.339 (3)	C10—C12	1.507 (3)
N2—C7	1.345 (3)	C11—H11	0.9500
			
O1—Cu1—O2^i^	168.33 (6)	C2—C3—H3	121.2
O1—Cu1—O4^ii^	89.67 (6)	C4—C3—H3	121.2
O2^i^—Cu1—O4^ii^	89.25 (7)	C5—C4—C3	118.87 (19)
O1—Cu1—O3^iii^	89.81 (6)	C5—C4—C6	121.06 (19)
O2^i^—Cu1—O3^iii^	88.91 (6)	C3—C4—C6	120.06 (19)
O4^ii^—Cu1—O3^iii^	168.37 (6)	N1—C5—C4	123.3 (2)
O1—Cu1—N2	99.05 (7)	N1—C5—H5	118.3
O2^i^—Cu1—N2	92.60 (6)	C4—C5—H5	118.3
O4^ii^—Cu1—N2	92.56 (6)	O2—C6—O1	126.63 (18)
O3^iii^—Cu1—N2	99.00 (6)	O2—C6—C4	116.34 (18)
O1—Cu1—Cu1^i^	85.28 (5)	O1—C6—C4	117.03 (18)
O2^i^—Cu1—Cu1^i^	83.08 (5)	N2—C7—C8	121.60 (18)
O4^ii^—Cu1—Cu1^i^	80.50 (4)	N2—C7—H7	119.2
O3^iii^—Cu1—Cu1^i^	87.88 (4)	C8—C7—H7	119.2
N2—Cu1—Cu1^i^	171.84 (5)	C7—C8—C9	120.71 (18)
C6—O1—Cu1	121.33 (13)	C7—C8—Br2	118.60 (15)
C6—O2—Cu1^i^	123.66 (13)	C9—C8—Br2	120.67 (16)
C12—O3—Cu1^iv^	117.81 (13)	C8—C9—C10	117.44 (19)
C12—O4—Cu1^v^	127.10 (14)	C8—C9—H9	121.3
C1—N1—C5	117.7 (2)	C10—C9—H9	121.3
C11—N2—C7	118.33 (18)	C9—C10—C11	119.06 (18)
C11—N2—Cu1	117.85 (14)	C9—C10—C12	122.30 (18)
C7—N2—Cu1	123.76 (14)	C11—C10—C12	118.57 (17)
N1—C1—C2	122.4 (2)	N2—C11—C10	122.86 (18)
N1—C1—H1	118.8	N2—C11—H11	118.6
C2—C1—H1	118.8	C10—C11—H11	118.6
C3—C2—C1	120.2 (2)	O3—C12—O4	126.62 (18)
C3—C2—Br1	120.35 (17)	O3—C12—C10	117.99 (18)
C1—C2—Br1	119.44 (15)	O4—C12—C10	115.38 (18)
C2—C3—C4	117.5 (2)		
			
O2^i^—Cu1—O1—C6	5.4 (4)	Cu1—O1—C6—C4	178.63 (13)
O4^ii^—Cu1—O1—C6	−79.30 (16)	C5—C4—C6—O2	−175.55 (19)
O3^iii^—Cu1—O1—C6	89.08 (16)	C3—C4—C6—O2	5.4 (3)
N2—Cu1—O1—C6	−171.84 (15)	C5—C4—C6—O1	4.8 (3)
Cu1^i^—Cu1—O1—C6	1.20 (15)	C3—C4—C6—O1	−174.31 (19)
O1—Cu1—N2—C11	−127.00 (15)	C11—N2—C7—C8	0.1 (3)
O2^i^—Cu1—N2—C11	53.57 (15)	Cu1—N2—C7—C8	177.04 (15)
O4^ii^—Cu1—N2—C11	142.93 (15)	N2—C7—C8—C9	0.1 (3)
O3^iii^—Cu1—N2—C11	−35.75 (15)	N2—C7—C8—Br2	178.81 (15)
O1—Cu1—N2—C7	56.05 (17)	C7—C8—C9—C10	0.1 (3)
O2^i^—Cu1—N2—C7	−123.39 (16)	Br2—C8—C9—C10	−178.61 (14)
O4^ii^—Cu1—N2—C7	−34.03 (16)	C8—C9—C10—C11	−0.4 (3)
O3^iii^—Cu1—N2—C7	147.30 (16)	C8—C9—C10—C12	176.54 (18)
C5—N1—C1—C2	−0.7 (3)	C7—N2—C11—C10	−0.5 (3)
N1—C1—C2—C3	0.8 (4)	Cu1—N2—C11—C10	−177.61 (15)
N1—C1—C2—Br1	−179.34 (18)	C9—C10—C11—N2	0.7 (3)
C1—C2—C3—C4	0.1 (3)	C12—C10—C11—N2	−176.43 (18)
Br1—C2—C3—C4	−179.72 (15)	Cu1^iv^—O3—C12—O4	4.0 (3)
C2—C3—C4—C5	−1.1 (3)	Cu1^iv^—O3—C12—C10	−174.39 (12)
C2—C3—C4—C6	178.03 (19)	Cu1^v^—O4—C12—O3	−3.7 (3)
C1—N1—C5—C4	−0.3 (3)	Cu1^v^—O4—C12—C10	174.71 (12)
C3—C4—C5—N1	1.2 (3)	C9—C10—C12—O3	1.3 (3)
C6—C4—C5—N1	−177.9 (2)	C11—C10—C12—O3	178.26 (18)
Cu1^i^—O2—C6—O1	−0.2 (3)	C9—C10—C12—O4	−177.28 (18)
Cu1^i^—O2—C6—C4	−179.81 (13)	C11—C10—C12—O4	−0.3 (3)
Cu1—O1—C6—O2	−1.0 (3)		

Symmetry codes: (i) −*x*+1, −*y*+2, −*z*; (ii) −*x*+3/2, *y*−1/2, −*z*+1/2; (iii) *x*−1/2, −*y*+5/2, *z*−1/2; (iv) *x*+1/2, −*y*+5/2, *z*+1/2; (v) −*x*+3/2, *y*+1/2, −*z*+1/2.
